# Stereospecificity of Oligonucleotide Interactions Revisited: No Evidence for Heterochiral Hybridization and Ribozyme/DNAzyme Activity

**DOI:** 10.1371/journal.pone.0115328

**Published:** 2015-02-13

**Authors:** Kai Hoehlig, Lucas Bethge, Sven Klussmann

**Affiliations:** NOXXON Pharma AG, Berlin, Germany; Beckman Research Institute of the City of Hope, UNITED STATES

## Abstract

A major challenge for the application of RNA- or DNA-oligonucleotides in biotechnology and molecular medicine is their susceptibility to abundant nucleases. One intriguing possibility to tackle this problem is the use of mirror-image (l-)oligonucleotides. For aptamers, this concept has successfully been applied to even develop therapeutic agents, so-called Spiegelmers. However, for technologies depending on RNA/RNA or RNA/DNA hybridization, like antisense or RNA interference, it has not been possible to use mirror-image oligonucleotides because Watson-Crick base pairing of complementary strands is (thought to be) stereospecific. Many scientists consider this a general principle if not a dogma. A recent publication proposing heterochiral Watson-Crick base pairing and sequence-specific hydrolysis of natural RNA by mirror-image ribozymes or DNAzymes (and *vice versa*) prompted us to systematically revisit the stereospecificity of oligonucleotides hybridization and catalytic activity. Using hyperchromicity measurements we demonstrate that hybridization only occurs among homochiral anti-parallel complementary oligonucleotide strands. As expected, achiral PNA hybridizes to RNA and DNA irrespective of their chirality. In functional assays we could not confirm an alleged heterochiral hydrolytic activity of ribozymes or DNAzymes. Our results confirm a strict stereospecificity of oligonucleotide hybridization and clearly argue against the possibility to use mirror-image oligonucleotides for gene silencing or antisense applications.

## Introduction

Stereospecificity of chemical and enzymatic reactions is a basic principle of the (bio)chemistry in our homochiral world [1]. The translational apparatus for ribosomal protein synthesis is a stereospecific process that only allows for the incorporation of l-amino acids into proteins. d-amino acids which are found in prokaryotic cell walls and also in peptides from higher organisms exclusively derive from post-translational modifications or ribosome-independent peptide synthesis [2]. Likewise, nucleic acids occur only in their d-configuration in nature. The absence of mirror-image l-nucleic acids (l-RNAs and l-DNAs) from living organisms makes them interesting tools for applications in diagnostics, sensoring or therapeutics since they are resistant against nucleolytic degradation and are immunologically passive [3,4].

The current use of oligonucleotides for therapeutic and other purposes is based on either of two general mechanisms: hybridization between nucleic acids through Watson-Crick base pairing or binding of oligonucleotides to other molecules through their three-dimensional shape. Watson-Crick base pairing is exploited in technologies such as antisense, RNA interference, exon skipping or ribozymes in which an oligonucleotide is hybridizing to a messenger RNA either to arrest its translation, to eliminate or to correct it. The ability of oligonucleotides to adopt complex three-dimensional structures and to bind to a target (similar to monoclonal antibodies) is the basis of aptamer technologies [5,6]. The question remains if and how mirror-image oligonucleotides can be introduced into these technologies to improve and extend their reach.

To make use of functional macromolecules of non-natural chirality (d-proteins or l-nucleic acids) requires some careful thoughts and also needs appropriate capabilities in synthetic chemistry since current molecular biology or biotechnology methods cannot be applied. A first breakthrough in this regard was published by Kent and co-workers in 1992 [7]. By synthesizing the HIV protease in the natural l- as well as the non-natural d-configuration the authors established what is called “reciprocal chiral substrate specificity”: The l-enzyme was able to cleave an l- but not a d-substrate whereas the corresponding d-enzyme cleaved only the d- but not the l-substrate. As expected, an achiral inhibitor blocked both the l- and the d-enzyme. The analyzed interactions, however, were kept in their “own homochiral world”, i.e. the l-polypeptide acted on the l-substrate whereas the exact mirror-image d-polypeptide acted on the d-substrate. The next logical question was how to implement new, artificial heterochiral interactions. This was first achieved with l-peptide binders that were selected to bind to a d-peptide target (the mirror-image of a naturally occurring l-peptide) using phage display. When the selected l-peptide binders were converted to their d-configuration, the resulting d-peptide binders recognized the natural l-peptide target [8]. The same principle was applied to oligonucleotides in order to generate biostable mirror-image (l-)aptamers, later called Spiegelmers (Spiegelmer is a registered trademark of NOXXON Pharma AG) [3]. For this purpose, d-nucleic acid libraries are screened against a non-natural mirror-image target (often a d-polypeptide). When the resulting d-aptamers are converted to l-aptamers, they will recognize the intended natural target (l-polypeptide) [9,10]. Recently, even an l-RNA aptamer was identified to bind to a structured d-RNA (TAR RNA) by selecting d-aptamers against the mirror-image l-TAR-RNA [11]. Importantly, also when looking at these heterochiral interactions, the reciprocal chiral specificity is maintained and there is no binding of aptamers and Spiegelmers to the enantiomer of their target, i.e. the target in its opposite configuration [8,12]. However, in case the selection target is achiral (e.g. theophylline), both the d- and l-configuration of an aptamer will bind with equal affinity [13].

In contrast to aptamers, it has not been possible to apply l-oligonucleotides to technologies that rely on the hybridization of complementary RNA or DNA strands. In the early 1990s there have been reports suggesting that l-oligonucleotides could hybridize to natural d-RNA or d-DNA and may therefore potentially be used for antisense applications. In these studies hybridization of l-homo-oligomers (oligo-l-U or -l-dA) to poly-d-T or poly-d-U was investigated and the formation of triple-stranded complexes was reported [14–17]. However, when Garbesi and co-workers later investigated the hybridization of mixed-sequence l-DNAs containing all four bases with complementary d-DNA or d-RNA molecules they did not find any evidence for heterochiral hybridization [18]. The authors concluded that “(…) l-DNAs have no future as wide scope antimessenger compounds (…)”. Thereafter, no further progress was reported on the use of l-oligonucleotides for antisense or related strategies.

Two decades later in early 2014, a recent publication now re-challenges the principle of stereospecific RNA and DNA hybridization by claiming that ribozymes and DNAzymes can hydrolyze RNA molecules irrespective of their chirality [19]. As the conclusions drawn from this study would have groundbreaking consequences not only for the field of oligonucleotide therapeutics but also for the general mechanisms of stereospecificity we sought to thoroughly re-evaluate the stereospecificity of oligonucleotide hybridization and ribozyme/DNAzyme activity systematically in independent experiments.

## Material and Methods

### Oligonucleotides

DNA/RNA-oligonucleotides were synthesized on an ABI-394 synthesizer using standard solid-phase phosphoramidite chemistry and workup procedures [20]. Phosphoramidites were purchased from Proligo (Hambug, Germany, l- and d-RNA phosphoramidites), Thermo Fisher Scientific (Milwaukee, USA, d-DNA phosphoramidites), ChemGenes (Wilmington, USA, l-DNA phosphoramidites) and Metkinen (Kuusisto, Finland, 5′-(6-fluorescein)phosphoramidite). Crude oligonucleotides were purified either by PAGE or ion exchange HPLC (IEX) followed by salt removal and subsequent size exclusion chromatography (NAP10, GE-Healthcare, Freiburg, Germany). Purity and identity was confirmed by IEX- and RP-HPLC and LC-MS (ESI-). PNA-oligonucleotides were synthesized by solid phase peptide synthesis following Fmoc/Bhoc strategy [21]. Fmoc/Bhoc protected PNA monomers were purchased from Link Technologies (Bellshill, UK). Synthesis commenced with 10 mg (2 μmol) Fmoc-protected amino-functionalized TentaGel R RAM solid support (200 μmol/g, Rapp Polymere, Tübingen, Germany). Manual solid phase synthesis was performed in 2 ml polyethylene syringe reactors that are equipped with a fritted disk applying a previously published protocol with minor alterations [22]. Purity and identity was confirmed by C8 RP-HPLC and LC-MS (ESI+).

### Strand hybridization

Hybridization of anti-parallel complementary and parallel complementary d- or l-RNA, d- or l-DNA and PNA strands was analyzed by measuring temperature-dependent hyperchromicity. Oligonucleotides (3 μM each) in 10 mM phosphate buffer (pH 7.4), 100 mM NaCl were heated to 95°C, cooled down to 25°C with 1°C/min, and heated up again to 95°C with 1°C/min. Absorption at 260 nm was measured in 0.5°C intervals. The melting temperature (T_m_) was calculated from the 1^st^ derivative of three 25°C to 95°C heating ramps. Experiments were performed in a Cary 100 spectrophotometer (Agilent, Böblingen, Germany). Data were analyzed using Prism 5.00 software (GraphPad, San Diego, California, USA). The following oligonucleotides were used:

d- or l-RNA template 5′-CUU CAA GUC CGC CA-3′

d- or l-DNA template 5′-CTT CAA GTC CGC CA-3′

Anti-parallel complementary d- or l-RNA 5′-UGG CGG ACU UGA AG-3′

Anti-parallel complementary d- or l-DNA 5′-TGG CGG ACT TGA AG-3′

Parallel complementary d- or l-RNA 5′-GAA GUU CAG GCG GU-3′

Parallel complementary d- or l-DNA 5′-GAA GTT CAG GCG GT-3′

Complementary PNA Ac-tgg cgg act tga ag-Gly-NH_2_

### RNA hydrolysis

Experiments were performed according to the protocols provided by Wyszko *et al*. [19]. Briefly, hydrolysis of 5′-fluorescein-labeled d- or l-RNA substrate (200 nM) by d- or l-hammerhead ribozyme or d- or l-DNAzyme (2 μM, unless otherwise indicated) was performed in 50 mM Tris (pH 7.5), 10 mM MgCl_2_. For control experiments, d- or l-RNA antisense oligonucleotides were added at 2 nM. Samples were heated to 95°C and cooled down to 25°C at 1°C/min. Hydrolysis was analyzed after 5 h incubation at 37°C using a denaturating 20% polyacrylamide, 7 M urea gel in TBE buffer. Gels were first stained with ethidium bromide staining and then documented under UV light. The following oligonucleotides were used:

d- or l-RNA substrate 5′-fluorescein-CUUCA AGUCC GCCA-3′

d- or l-ribozyme 5′-UGGCG CUGAU GAGGC CGAAA GGCCG AAACU UGA-3′

d- or l-DNAzyme 5′-GGCGG AGGCT AGCTA CAACG ATTGA AG-3′

d- or l-RNA antisense 5′-UCAAG UUUCG GCCUU UCGGCC UCAUC AGCG CCA-3′

## Results

### Oligonulceotide hybridization is strictly stereospecific

**Fig 1:**
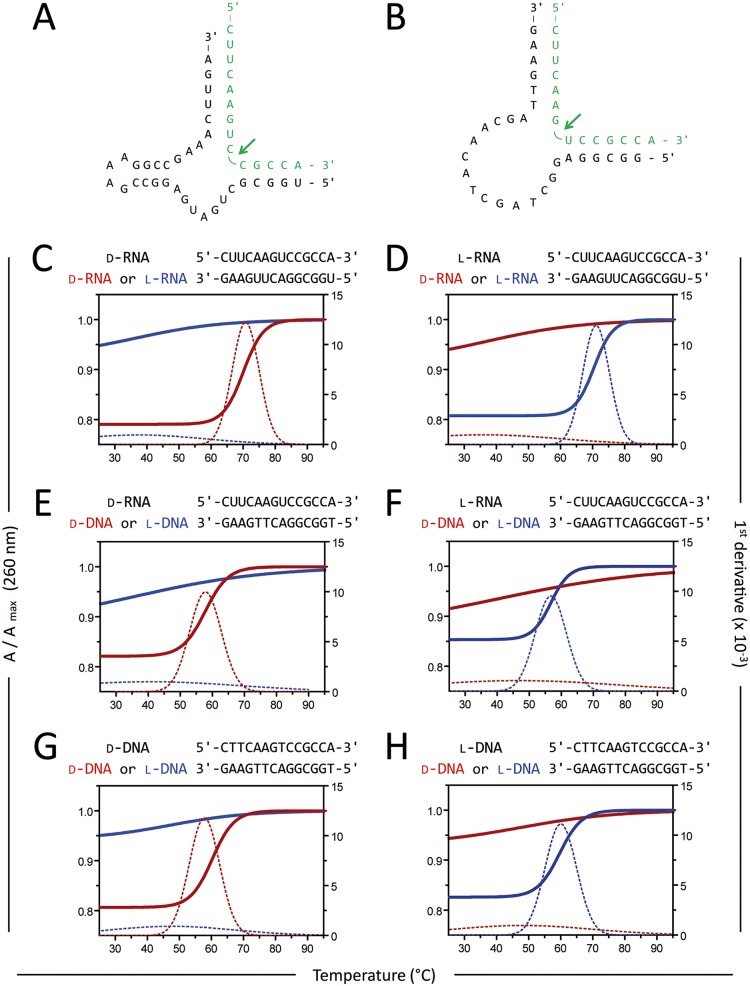
Hybridization of anti-parallel complementary oligonucleotides is stereospecific. Schematic representation of (A) hammerhead ribozyme and (B) DNAzyme in complex with the target RNA sequence (green). Hydrolysis sites are indicated by arrows. To analyze (potential) hybridization of enantiomeric, anti-parallel complementary oligonucleotides (3 μM each in 10 mM phosphate buffer, pH 7.4, 100 mM NaCl) temperature-dependent hyperchromicity at 260 nm was measured. (C) d-RNA and (D) l-RNA with anti-parallel complementary d-RNA (red) and l-RNA (blue). (E) d-RNA and (F) l-RNA with anti-parallel complementary d-DNA (red) and l-DNA (blue). (G) d-DNA and (H) l-DNA with anti-parallel complementary d-DNA (red) and l-DNA (blue). Mean of three melting ramps (25°C to 95°C) is given as normalized absorption A / Amax at 260 nm. First derivative is shown as dotted line. Data are representative of three independent experiments.

According to the current understanding, ribozyme/DNAzyme mediated hydrolysis of an RNA target molecule requires the hybridization of the target sequence with anti-parallel, complementary recognition sites of the ribozyme (Fig. 1A) or DNAzyme (Fig. 1B). To explore whether this premise would still be true for heterochiral RNA/ribozyme or RNA/DNAzyme interactions we used enantiomeric oligonucleotides complementary to the 14 nt RNA target sequence and firstly measured temperature-dependent hyperchromicity. In a homochiral setting, anti-parallel, complementary d-RNA/d-RNA (Fig. 1C) and l-RNA/l-RNA (Fig. 1D) molecules showed comparable melting profiles with T_m_ = 70.9 ± 0.1°C and T_m_ = 70.8 ± 0.1°C for (mean ± sem of n = 3), respectively. In contrast, no sharp hyperchromicity transition was observed when heterochiral, anti-parallel, complementary RNA strands were tested (Fig. 1C, 1D). Basically, the same results but with reduced melting temperatures were observed for RNA/DNA and DNA/DNA duplexes. Homochiral, anti-parallel, complementary d-RNA/d-DNA (Fig. 1E) and l-RNA/l-DNA (Fig. 1F) strands displayed melting temperatures of T_m_ = 57.8 ± 0.1°C and T_m_ = 56.9 ± 0.1°C (mean ± sem of n = 3), respectively, and d-DNA/d-DNA (Fig. 1G) and l-DNA/l-DNA (Fig. 1H) duplexes showed melting temperatures of T_m_ = 60.4 ± 0.1°C and T_m_ = 60.0 ± 0.2°C (mean ± sem of n = 3), respectively. Significant hyperchromicity indicating hybridization of heterochiral strands could not be detected for any of the tested examples (Fig. 1C-1H). Furthermore, we investigated if heterochiral oligonucleotides would interact in parallel, complementary orientation as previously suggested [23]. Hyperchromicity transition was neither observed for homochiral nor heterochiral, parallel complementary RNA/RNA (S1A, S1B Fig.), RNA/DNA (S1C, S1D Fig.) or DNA/DNA (S1E, S1F Fig.) strands.

**Fig 2:**
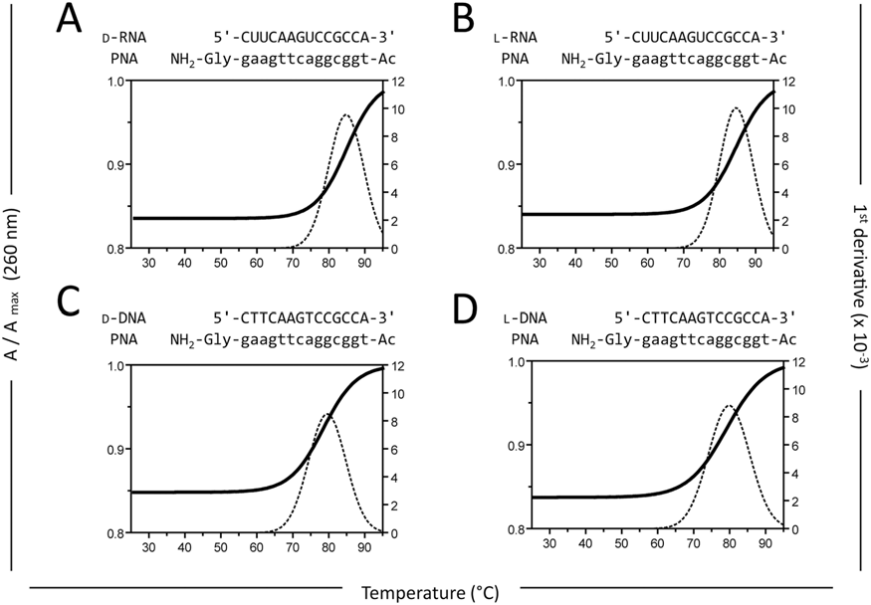
Achiral PNA hybridizes to both d- and l-RNA/DNA. Hybridization of achiral PNA and anti-parallel complementary (A) d-RNA, (B) l-RNA, (C) d-DNA, and (D) l-DNA (3 μM each in 10 mM phosphate buffer, pH 7.4, 100 mM NaCl) was analyzed by measuring temperature-dependent hyperchromicity at 260 nm. Mean of three melting ramps (25°C to 95°C) is given as normalized absorption A / A_max_ at 260 nm. First derivative is shown as dotted line. Data are representative of three independent experiments.

To finally complete the hybridization experiments, we analyzed the interaction of achiral peptide nucleic acid (PNA) with RNA and DNA oligonucleotides of either chirality. As expected, PNA hybridization was independent of the chirality: the hybridization of d-RNA (Fig. 2A) and l-RNA (Fig. 2B) to anti-parallel complementary PNA yielded almost identical melting temperatures of T_m_ = 84.4 ± 0.1°C and T_m_ = 84.3 ± 0.2°C (mean ± sem of n = 3), respectively. A similar picture was observed for d-DNA (Fig. 2C) and l-DNA (Fig. 2D) interacting with anti-parallel complementary PNA. Here the melting temperatures were slightly lower: T_m_ = 80.1 ± 0.3°C and T_m_ = 79.6 ± 0.1°C (mean ± sem of n = 3), respectively.

### Ribozyme and DNAzyme activity is strictly stereospecific

**Fig 3:**
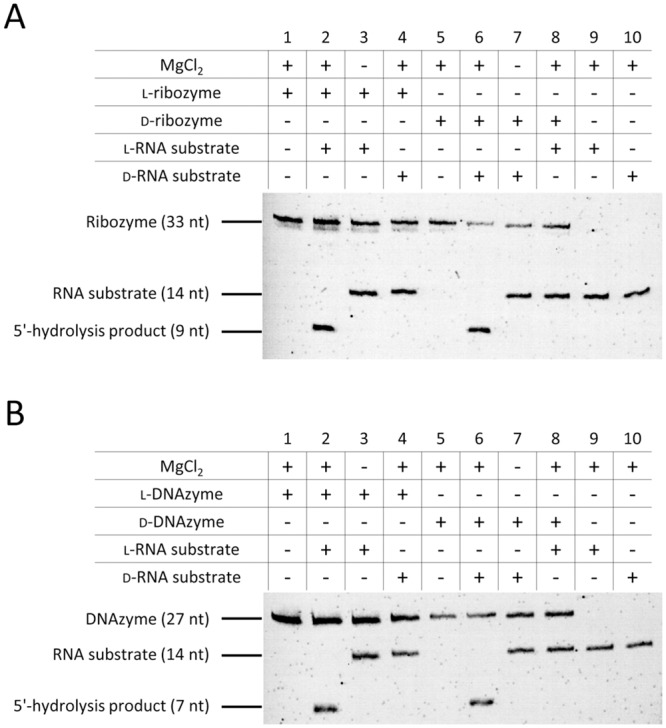
Stereospecificity of ribozyme and DNAzyme activity. 5′-fluorescein-labeled d- or l-RNA substrate (200 nM) was incubated with (A) d- or l-hammerhead ribozyme (2 μM) or (B) d- or l-DNAzyme (2 μM) in 50 mM Tris (pH 7.5), 10 mM MgCl_2_ for 5 h at 37°C. RNA substrate and its 5′-hydrolysis products were visualized via a 5′-fluorescein tag. Ribozymes and DNAzymes were stained with ethidium bromide. Data is representative of three independent experiments.

For a functional analysis we tested the stereospecificity of RNA hydrolysis using a hammerhead ribozyme (Fig. 1A and Fig. 3A) and a DNAzyme (Fig. 1B and Fig. 3B) following the protocol published by Wyszko *et al*. [19]. Homochirality of RNA substrate and enzymatically active nucleic acid (ribozyme or DNAzyme) resulted in complete cleavage (Fig. 3A and 3B, lanes 2 and 6). In all cases, the homochiral cleavage is MgCl_2_-dependent (Fig. 3A and 3B, lanes 3 and 7). In contrast, no cleavage was observed when RNA substrates were incubated with heterochiral ribozyme or DNAzyme (Fig. 3A and 3B, lanes 4 and 8). The identity of the cleavage products was confirmed by mass spectrometry (S2 Fig.). Thus, in agreement with the hybridization data, we can show that the activity of ribozyme and DNAzyme is strictly stereospecific.

### Experiments focusing on stereospecificity need to be tightly controlled

**Fig 4:**
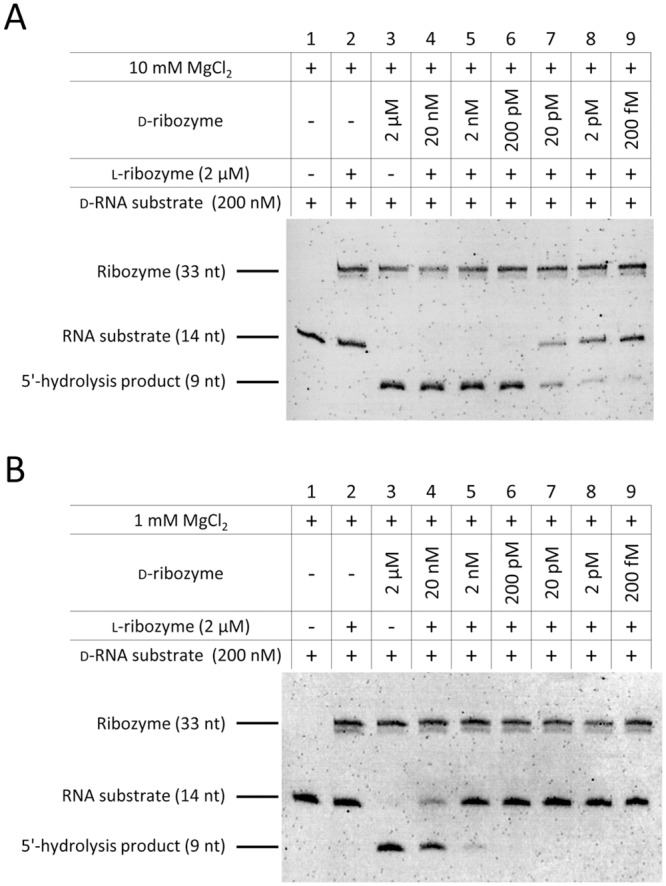
Ribozyme cleavage is prone to enantiomeric contaminations. l-hammerhead ribozyme solution (2 μM) was artificially contaminated with enantiomeric d-ribozyme (20 nM–200 fM) and cleavage of d-RNA substrate was analyzed after 5 h at 37°C. Assays were performed in 50 mM Tris, pH 7.5 in the presence of (A) 10 mM MgCl_2_ and (B) 1 mM MgCl_2_. RNA substrate and its 5′-hydrolysis products were visualized via a 5′-fluorescein tag. Ribozymes and DNAzymes were stained with ethidium bromide. Data is representative of two independent experiments.

Enzymatic RNA hydrolysis by ribozymes is a highly efficient process which requires the need for careful planning and tight experimental control, especially in stereospecificity studies. For example, 0.01% of enantiomeric d-ribozyme (200 pM) contaminating an l-ribozyme solution (2 μM) creates a false positive “heterochiral” cleavage product that is indistinguishable from that generated by real homochiral RNA hydrolysis (Fig. 4, lanes 6 and 3, respectively). A first measure to reduce the risk of generating artefacts can be a reduction of the MgCl_2_ concentration from 10 mM to a physiological level of 1 mM. Under these conditions, a 0.1% contamination with d-ribozyme (2 nM) is largely tolerated (Fig. 4B, lane 5).

**Fig 5:**
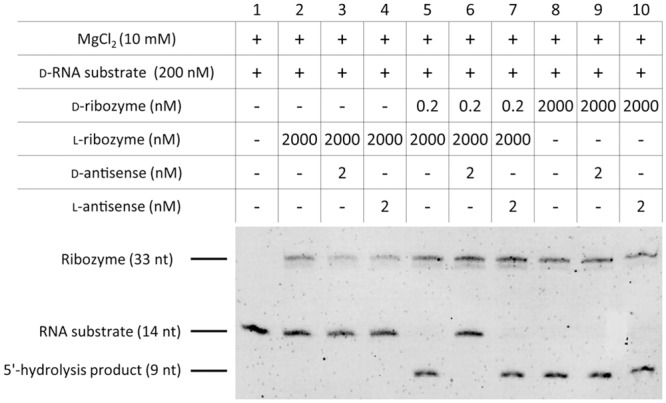
Antisense probes for the experimental control of stereospecificity. l-ribozyme solution (2 μM) was artificially contaminated with 0.01% enantiomeric d-ribozyme (0.2 nM) and cleavage of d-RNA substrate was analyzed after 5 h at 37°C. d- and l-antisense probes (2 nM) were added to control stereospecificity. Assays were performed in 50 mM Tris, pH 7.5, 10 mM MgCl_2_. RNA substrate and its 5′-hydrolysis products were visualized via a 5′-fluorescein tag. Ribozymes and DNAzymes were stained with ethidium bromide. Data is representative of two independent experiments.

Since the differentiation between l- and d-configured oligonucleotides with standard molecular biology methods and equipment is challenging, careful control strategies have to be established to uncover false-positive results. A rather simple way to control this type of assays is the use of enantiomeric antisense probes. As discussed above, contamination of an l-ribozyme with as little as 0.01% d-ribozyme creates a pretended “heterochiral” hydrolysis product (Fig. 5, lane 5) which is indistinguishable from the d-homochiral product (Fig. 5, lane 8). Addition of 0.1% d-antisense probe completely blocks the alleged “heterochiral” cleavage reaction (Fig. 5, lane 6) while leaving the d-homochiral unaffected (Fig. 5, lane 9). Importantly, this blockade is again stereospecific as addition of 0.1% l-antisense has no impact (Fig. 5, lane 7).

## Discussion

We investigated the stereospecificity of oligonucleotide hybridization and RNA hydrolysis by enzymatically active nucleic acids and show that both are strictly limited to homochiral oligonucleotides. In a first paper in 2013, Wyszko *et al*. described that ribozymes and DNAzymes composed of l-nucleic acids are able to cleave l-RNA substrates [24]. This observation is in agreement with the basic concept of chirality that enantiomeric molecules must have identical chemical properties. Furthermore, the outcome could be deduced from previous data generated with either d-proteins or l-nucleic acids [3,7,8,25]. As expected, we were able to confirm that homochiral hybridization of anti-parallel complementary strands as well as homochiral RNA cleavage by either a ribozyme or a DNAzyme occurs with the same thermodynamics and efficiency with either l- or d-configured oligonucleotides.

In a second paper published early 2014, Wyszko *et al*. went one step further claiming that ribozymes and DNAzymes are also able to sequence-specifically hydrolyze RNA in a heterochiral manner [19]. In a structural model the authors propose that this phenomenon is based on helices formed by Watson-Crick base pairing between heterochiral strands. Since these results contradict the current understanding, we thoroughly investigated the hybridization of heterochiral oligonucleotides by hyperchromicity measurements. We were not able to detect any specific hybridization between oligonucleotides of different configuration (Fig. 1). Also in line with the basic principles of stereospecific recognition, PNA oligomers as achiral substances sequence-specifically hybridize to RNA and DNA irrespective of their chirality (Fig. 2) [26].

Consequently, in tightly controlled experiments we were also not able to reproduce any hydrolytic activity of ribozymes or DNAzyms towards RNA substrates of different chirality (Fig. 3). Since ribozyme/DNAzyme-mediated RNA hydrolysis is a very effective catalytic process we experienced in our own hands that (under the given experimental conditions) minimal contaminations of the ‘unwanted’ ribozyme/DNAzyme may easily lead to false positive results. In this context it needs to be pointed out that the data presented by Wyszko *et al*. are also not consistent with the principle of identical chemical properties of enantiomers. Cleavage of d-RNA by the l-riboyzme is much less efficient than l-RNA cleavage by the d-riboyzme and furthermore only the l- but not d-DNAzyme is reported to be capable of heterochiral activity [19]. If sequence-specific heterochiral cleavage would indeed take place hydrolysis would need to occur at the same efficiency for the d-/l- and l-/d-pairs of target and ribozyme/DNAzyme.

Thus, we strongly recommend when investigating the stereospecificity of enzymatic processes to perform this kind of experiments at more physiological conditions (reduced MgCl_2_, no ribozyme/DNAzyme excess) and, what is most important, to include appropriate controls. An example for such a control experiment is the use of antisense probes that may help to discriminate between a *de facto* cleavage and an artificial cleavage product derived from contaminations (Fig. 5).

We have systematically investigated the hybridization behavior of oligonucleotides displaying the d- and l-configuration. The results confirm that sequence-specific (Watson-Crick-based) hybridization does not occur between oligonucleotides of different chirality. Consequently, heterochiral ribozyme/DNAzyme cleavage of RNA molecules is not possible as long as the mechanisms of the catalytically active nucleic acids rely on hybridization to their respective substrates. However, the powerful techniques of *in vitro* selection and *in vitro* evolution are able to deliver mirror-image oligonucleotide candidates [27] that cannot only recognize an RNA substrate by Watson-Crick-independent tertiary interactions [11] but may eventually even be able to catalytically modulate a substrate RNA. In this regard, a *cross-chiral RNA polymerase ribozyme* has recently been reported [28].

## Supporting Information

S1 Fig No hybridization of parallel complementary oligonucleotides. Hybridization of enantiomeric, parallel complementary oligonucleotides (3 μM each in 10 mM phosphate buffer, pH 7.4, 100 mM NaCl) was followed by measuring temperature-dependent hyperchromicity at 260 nm. (A) d-RNA and (B) l-RNA with parallel complementary d-RNA (red) and l-RNA (blue). (C) d-RNA and (D) l-RNA with parallel complementary d-DNA (red) and l-DNA (blue). (E) d-DNA and (F) l-DNA with parallel complementary d-DNA (red) and l-DNA (blue). Mean of three melting ramps (25°C to 95°C) is given as normalized absorption A / A_max_ at 260 nm. First derivative is shown as dotted line. Data is representative of two independent experiments.
(PDF)

S2 Fig Confirmation of hydrolysis products by mass spectrometry. Homochiral hydrolysis of 5′-fluorescein-labeled RNA substrate (5 μM) by (A) was incubated with (A) l-hammerhead ribozyme (50 μM), (B) l-DNAzyme (2 μM), (C) d-hammerhead ribozyme (50 μM) or (D) d-DNAzyme (50 μM) in 50 mM Tris (pH 7.5) (samples 2, 4, 6, 8) or in 50 mM Tris (pH 7.5), 10 mM MgCl_2_ (samples 1, 3, 5, 7) for 5 h at 37°C. Samples were analyzed by PAGE and LC-MS (ESI-).
(PDF)
