# Supplementary material for: Stereospecificity of Oligonucleotide Interactions Revisited: No Evidence for Heterochiral Hybridization and Ribozyme/DNAzyme Activity
Source: PLoS One. 2015 Feb 13;10(2):e0115328. doi: 10.1371/journal.pone.0115328 (PMC4334536; doi:10.1371/journal.pone.0115328)
Supplement: S1 Fig — Hybridization of enantiomeric, parallel complementary oligonucleotides (3 μM each in 10 mM phosphate buffer, pH 7.4, 100 mM NaCl) was followed by measuring temperature-dependent hyperchromicity at 260 nm. (A) d-RNA and (B) l-RNA with parallel complementary d-RNA (red) and l-RNA (blue). (C) d-RNA and (D) l-RNA with parallel complementary d-DNA (red) and l-DNA (blue). (E) d-DNA and (F) l-DNA with parallel complementary d-DNA (red) and l-DNA (blue). Mean of three melting ramps (25°C to 95°C) is given as normalized absorption A / Amax at 260 nm. First derivative is shown as dotted line. Data is representative of two independent experiments. (PDF) [file pone.0115328.s001.pdf]

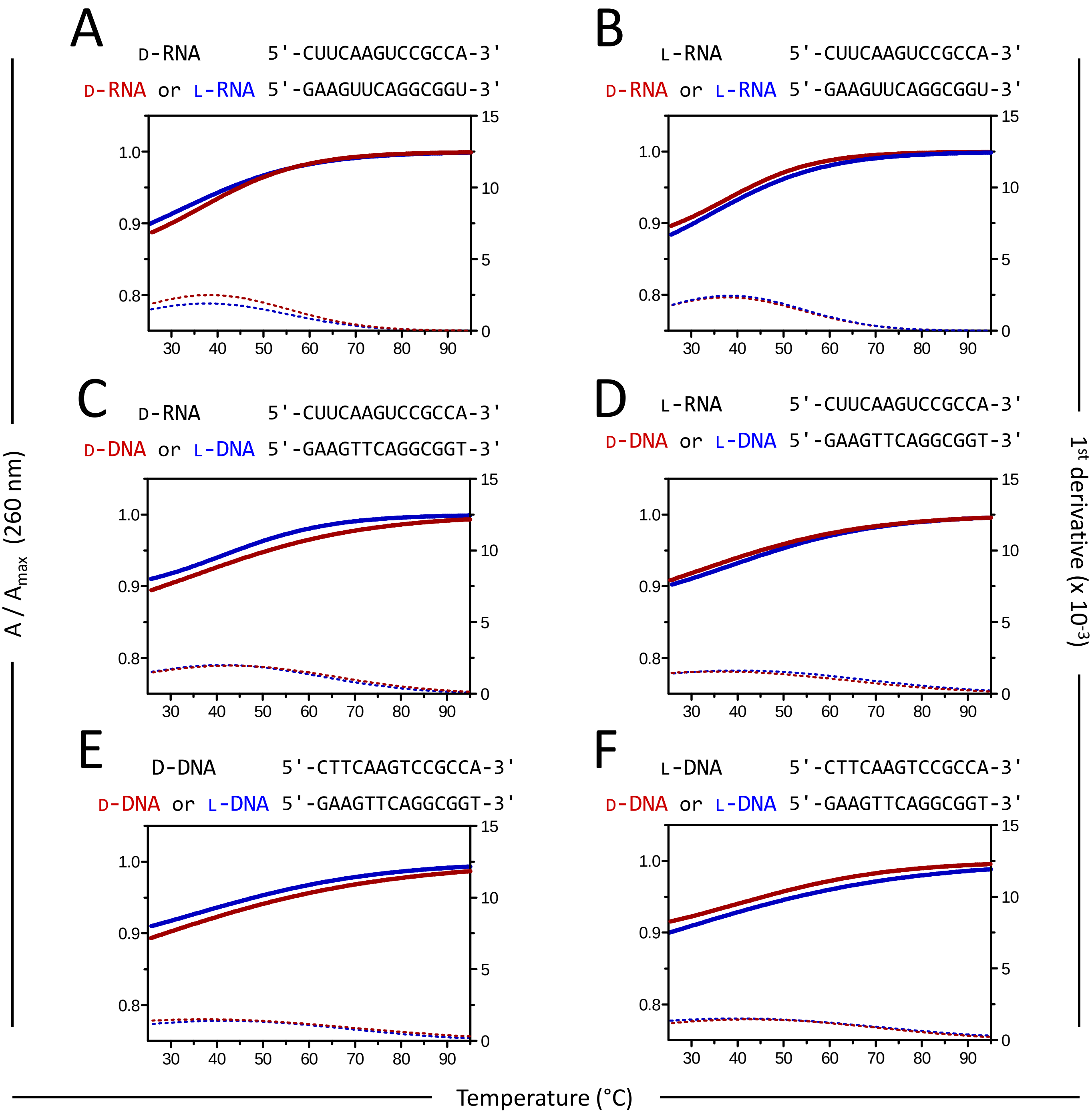
A
D-RNA 5'-CUUCAAGUCCGCCA-3'
D-RNA or L-RNA 5'-GAAGUUCAGGCGGU-3'
B
L-RNA 5'-CUUCAAGUCCGCCA-3'
D-RNA or L-RNA 5'-GAAGUUCAGGCGGU-3'
C
D-RNA 5'-CUUCAAGUCCGCCA-3'
D-DNA or L-DNA 5'-GAAGTTCAGGCGGT-3'
D
L-RNA 5'-CUUCAAGUCCGCCA-3'
D-DNA or L-DNA 5'-GAAGTTCAGGCGGT-3'
E
D-DNA 5'-CTTCAAGTCCGCCA-3'
D-DNA or L-DNA 5'-GAAGTTCAGGCGGT-3'
F
L-DNA 5'-CTTCAAGTCCGCCA-3'
D-DNA or L-DNA 5'-GAAGTTCAGGCGGT-3'
A / Amax (260 nm)
1st derivative (x 10-3)
Temperature (°C)
