# Supplementary figures and images for: Stereospecificity of Oligonucleotide Interactions Revisited: No Evidence for Heterochiral Hybridization and Ribozyme/DNAzyme Activity
Source: PLoS One. 2015 Feb 13;10(2):e0115328. doi: 10.1371/journal.pone.0115328 (PMC4334536; doi:10.1371/journal.pone.0115328)

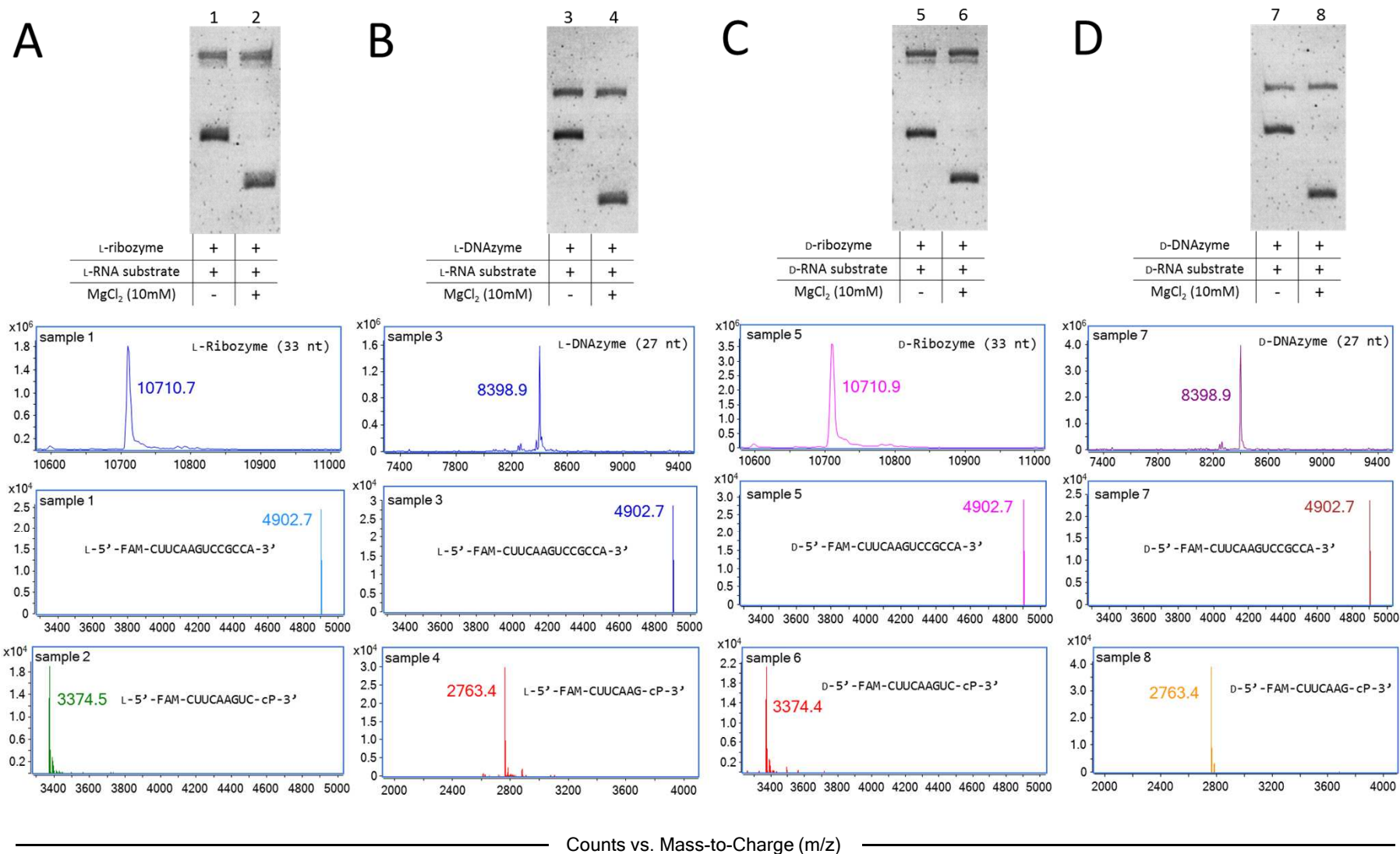

Supplement: S2 Fig — Homochiral hydrolysis of 5′-fluorescein-labeled RNA substrate (5 μM) by (A) was incubated with (A) l-hammerhead ribozyme (50 μM), (B) l-DNAzyme (2 μM), (C) d-hammerhead ribozyme (50 μM) or (D) d-DNAzyme (50 μM) in 50 mM Tris (pH 7.5) (samples 2, 4, 6, 8) or in 50 mM Tris (pH 7.5), 10 mM MgCl2 (samples 1, 3, 5, 7) for 5 h at 37°C. Samples were analyzed by PAGE and LC-MS (ESI-). (PDF) [file pone.0115328.s002.pdf]
